# Differential role of CSF alpha-synuclein species, tau, and Aβ42 in Parkinson's Disease

**DOI:** 10.3389/fnagi.2014.00053

**Published:** 2014-03-31

**Authors:** Lucilla Parnetti, Lucia Farotti, Paolo Eusebi, Davide Chiasserini, Claudia De Carlo, David Giannandrea, Nicola Salvadori, Viviana Lisetti, Nicola Tambasco, Aroldo Rossi, Nour K. Majbour, Omar El-Agnaf, Paolo Calabresi

**Affiliations:** ^1^Clinica Neurologica, Università degli Studi di PerugiaPerugia, Italy; ^2^Dipartimento di Epidemiologia, Regione UmbriaPerugia, Italy; ^3^Dipartimento di Scienze Farmaceutiche, Sezione di Biochimica, Università degli Studi di PerugiaPerugia, Italy; ^4^Department of Biochemistry, Faculty of Medicine and Health Sciences, United Arab Emirates UniversityAl Ain, United Arab Emirates; ^5^Faculty of Medicine, King Abdulaziz UniversityJeddah, Saudi Arabia; ^6^IRCCS Fondazione S. LuciaRoma, Italy

**Keywords:** cerebrospinal fluid biomarkers, alpha synuclein, total tau, phosphorylated tau, Aβ_42_, Parkinson's disease, cognitive decline

## Abstract

There is a great interest in developing cerebrospinal fluid (CSF) biomarkers for diagnosis and prognosis of Parkinson's disease (PD). CSF alpha synuclein (α-syn) species, namely total and oligomeric α-syn (t-α-syn and o-α-syn), have shown to be of help for PD diagnosis. Preliminary evidences show that the combination of CSF t-α-syn and classical Alzheimer's disease (AD) biomarkers—β-amyloid 1–42 (Aβ_42_), total tau (t-tau), phosphorylated tau (p-tau)—differentiate PD patients from controls, and that reduced levels of Aβ_42_ represent a predictive factor for development of cognitive deterioration in PD. In this prospective study carried out in 44 PD patients and 25 neurological controls we wanted to verify whether the combination of CSF α-synuclein species—t-α-syn and o-α-syn—and classical AD biomarkers may help in differentiating PD from neurological controls, and if these biomarkers may predict cognitive decline. The median of follow-up duration was 3 years (range: 2–6 years). Mini Mental State Examination (MMSE) and Montreal Cognitive Assessment (MoCA) were used for monitoring cognitive changes along time, being administered once a year. Oligo/total α-syn ratio (o/t-α-syn ratio) confirmed its diagnostic value, significantly contributing to the discrimination of PD from neurological controls. A greater diagnostic accuracy was reached when combining o/t-α-syn and Aβ_42_/tau ratios (Sens = 0.70, Spec = 0.84, AUC = 0.82; PPV = 0.89, NPV = 0.62, LR+ = 4.40, DOR = 12.52). Low CSF Aβ_42_ level was associated with a higher rate of MMSE and MoCA decline, confirming its role as independent predictive factor for cognitive decline in PD. None of the other biomarkers assessed (t-tau, p-tau, t-α-syn and o-α-syn) showed to have prognostic value. We conclude that combination of CSF o/t-α-syn and Aβ_42_/tau ratios improve the diagnostic accuracy of PD. PD patients showing low CSF Aβ_42_ levels at baseline are more prone to develop cognitive decline.

## Introduction

Parkinson's disease (PD) is a common neurodegenerative disorder evolving, in a substantial proportion of patients, to dementia. Although it is defined as a typical movement disorder and its diagnosis is mainly based on motor-related clinical criteria, other functional domains are also involved. Accordingly, post-mortem findings of alpha-synuclein (α-syn) pathology—the histopathologic hallmark of PD—show that the involvement of the dorsal motor nucleus of the vagal nerve and the olfactory bulb takes place much earlier before midbrain involvement (Braak et al., 2003).

PD is a complex neurodegenerative disorder in which many different pathophysiological processes take place, such as protein aggregation, oxidative damage and lysosomal dysfunction (Parnetti et al., 2013). Concomitant pathologies (i.e., Alzheimer and Lewy bodies pathologies) resulting from the mutual interaction between Aβ_42_, tau and α-syn during the course of the disease have major role in the neuropathological processes underlying dementia in PD (Tsigelny et al., 2008; Ciaccioli et al., 2013). In PD the spread of fibrillar α-syn pathology from the brainstem to limbic and neocortical structures, and the cortical deposition of β-amyloid plaques, represent major events (Compta et al., 2011; Irwin et al., 2012). Co-occurrence of tau and α-syn pathology has been found in neurons of brains affected by tauopathies and synucleinopathies, including PD (Vekrellis et al., 2011). Also, α-syn causes aggregation and polymerization of tau, which then induces the formation of intracellular amyloid-tau inclusions (Waxman and Giasson, 2011).

Since molecular changes in the brain are reflected in cerebrospinal fluid (CSF) composition, the CSF represents an ideal source for biomarkers of different pathophysiological processes characterizing the early phases of the disease, when the clinical diagnosis is more challenging. For example, Aβ_42_, total tau (t-tau) and phosphorylated tau (p-tau) are state markers of Alzheimer's disease (AD), as they reliably reflect AD pathology also in pre-dementia phases. This knowledge has been translated into operational diagnostic criteria (Dubois et al., 2010). Analogously, there is great interest for improving early diagnosis in PD, hopefully in the pre-motor phases, as well as for detecting PD patients at risk of dementia.

Currently, detection of reliable CSF biomarkers for PD is under intensive investigation. Several recent studies have explored the potential use of CSF total α-syn (t-α-syn) as a putative PD biomarker. A clear trend of lower CSF t-α-syn levels in PD and other synucleinopathies has been consistently reported (Tokuda et al., 2006; Noguchi-Shinohara et al., 2009; Spies et al., 2009; Hong et al., 2010; Mollenhauer et al., 2011; Parnetti et al., 2011; Aerts et al., 2012; Tateno et al., 2012), although with a large overlap between the PD and control groups (Noguchi-Shinohara et al., 2009; Spies et al., 2011). Therefore, the measurement of CSF t-α-syn doesn't seem to have enough specificity to correctly discriminate patients with synucleinopathies from normal individuals or other neurodegenerative diseases. The measurement in CSF of other α-syn species, namely soluble oligomers (o-α-syn) has improved the discrimination between PD and other diseases. O-α-syn levels are elevated in brain homogenates in PD and dementia with Lewy bodies compared with normal brains (Paleologou et al., 2009) suggesting a role for o-α-syn in PD pathogenesis. CSF o-α-syn levels and o/t-α-syn ratio have been consistently found to be significantly higher in PD patients as compared to other neurological disorders, with good sensitivity and specificity, as confirmed in independent reports (Tokuda et al., 2010; Park et al., 2011; Sierks et al., 2011; Parnetti et al., 2014). The high risk of cognitive impairment in PD also calls for biomarkers able to predict dementia onset. Many studies have focused on classical AD CSF biomarkers (Parnetti et al., 2008; Compta et al., 2009; Alves et al., 2010; Montine et al., 2010; Siderowf et al., 2010; Leverenz et al., 2011) and most of them have identified the reduction of CSF Aβ_42_ levels as a prognostic factor for cognitive impairment in PD. Data on the possible relationship between α-syn species and the risk of dementia in PD are still scanty.

In this study we evaluated both the diagnostic accuracy and the capability in predicting cognitive decline of CSF AD biomarkers (Aβ_42_, t-tau, p-tau and Aβ_42_/t-tau ratio) and α-syn species (t-α-syn, o-α-syn and o/t-α-syn ratio) in PD patients and neurological controls with a median follow up duration of 3 years.

## Materials and methods

### Patients

The subjects included in this study (44 PD, 25 neurological controls) were consecutively recruited between 2007 and 2011 and followed-up. They underwent a baseline clinical examination by experienced neurologists, detailed neuropsychological testing, blood chemistry, neuroimaging (computed tomography and/or magnetic resonance imaging), and lumbar puncture. CSF was collected according to the hospital standard protocol and with the local ethical committee approval, after informed written consent was given by the patient. All PD patients fulfilled the United Kingdom Parkinson's Disease Society Brain Bank clinical diagnostic criteria. All of them were treated with L-DOPA and the great majority (39 out of 44) were also taking DA-agonists. PD patients had good control of motor symptoms (mean UPRDS-III 25.39 ± 12.97), and were functionally independent or minimally dependent (Hoehn and Yahr, 1–2.5). Disease duration was calculated from the onset of the first motor symptoms to the time of lumbar puncture. As a control group, 25 cognitively normal age-matched subjects who underwent lumbar puncture as a part of diagnostic work up for other neurological conditions (OND) were recruited to our study. The OND group included: primary headache (*n* = 15), postural instability (*n* = 3), seizures (*n* = 2) and polyneuropathy (*n* = 5). Follow-up visits included clinical examination and neuropsychological testing carried out yearly by means of MMSE (Folstein et al., 1975), a popular screening tool mostly measuring cortical functions with special attention to memory, and MoCA (Gill et al., 2008), another screening tool assessing executive functions.

### CSF sampling

Lumbar puncture was performed between 8:00 and 10:00 A.M., after an overnight fast. CSF (10 mL) was collected in sterile polypropylene tubes, centrifuged for 10 min at 2000× g, and 0.5-mL aliquots were immediately frozen at −80°C. None of the samples was contaminated by blood during the procedure (samples showing an erythrocyte count >500/mm^3^ were not included in the study). CSF Aβ_42_, t-tau and p-tau were measured with ELISA assays (Innotest β Amyloid 1–42, hTAU-Ag, p-TAU 181 Ag; Innogenetics NV, Gent, Belgium, now Fujirebio). For CSF α-syn, 0.5 mL samples were thawed on ice and then divided into aliquots of 110 μL in siliconized tubes containing a cocktail of protease inhibitors, including AEBSF, aprotinin, E-64, EDTA, and leupeptin (Calbiochem-Novabiochem Corporation, San Diego, CA), and 0.05% Tween 20, and the samples were then stored at −80°C until used in the immunoassay for α-syn. While CSF t-α-syn is expressed as ng/mL, CSF o/t-α-syn ratio is expressed in CPS (counts per second). All the samples were obtained at the Section of Neurology, Perugia General Hospital, according to the protocol approved by the Regional Ethical Committee (Prot. N. 19369/AV), after informed written consent was obtained.

### Immunoassay for α-synuclein in CSF

Total and oligomeric CSF α-syn were measured as previously reported (Tokuda et al., 2006). Briefly, for CSF t-α-syn anti-human α-syn monoclonal antibody (clone Syn211) (Santa Cruz Biotechnology, USA) was used for capturing while the anti-human α-syn polyclonal antibody FL-140 (Santa Cruz Biotechnology, USA) was used for antigen detection. The standard curve for the ELISA assay was constructed using recombinant human α-syn solution at different concentrations diluted in blocking buffer. For α-syn oligomers, the antibody clone Syn211 was used for capturing, while biotinylated Syn211 (Santa Cruz Biotechnology, USA) was used for antigen detection. The plate was incubated with 50 μ L/well of ExtrAvidin-Peroxidase (Sigma-Aldrich, UK) and with the enhanced chemiluminescent substrate. For both immunoassays, the samples were screened in blind fashion and were randomly tested. A series of internal controls were run to check for run-to-run variations.

### Data analysis

Statistical analyses were performed using R software v. 2.15 (R Core Team, 2013). Continuous variables were described by median and ranges since data distributions were skewed. Correlations were calculated using Spearman's Rho (r). Kruskal-Wallis test was initially used for comparisons between the two diagnostic groups (*p* < 0.05). The accuracy of the diagnostic value of the biomarkers was assessed by area under the curve (AUC) of the receiver operating characteristic (ROC) curve (Robin et al., 2011; Eusebi, 2013). Cut-off values were calculated using sensitivity and specificity values that maximized Youden's index (sensitivity + specificity − 1). For evaluating the role of multiple biomarkers a multivariable logistic regression approach was used. With the aim to find the best predictors of PD to be included in the final model, we considered all the CSF enzyme activities which had already shown significant differences between OND and PD groups after the univariate analysis.

Multivariate linear regression analysis was used for analyzing the biomarker role in predicting cognitive decline. Change in MMSE and MoCA score were considered as dependent variables. Multiple imputations for missing values were performed in multivariable analyses (Rubin, 1987). Missing data were filled in five times to generate five complete data sets. The completed datasets were analyzed by using the mixed-effects model and the results were combined for the inference.

## Results

### Descriptive analysis

Demographic data, clinical features, and biomarkers values are listed in Table 1. As expected, no significant difference between PD and OND groups was found with respect to age, gender, and follow-up duration. Both MMSE and MoCA scores were significantly lower at baseline (*p* = 0.009 and *p* = 0.025, respectively) and after follow-up (*p* = 0.004 and *p* < 0.001, respectively). Follow-up observations refer to the last visit carried out.

**Table 1:** **Demographic data and clinical features for OND and PD**.

	**OND**	**PD**	***p*-value**
*N*	25	44	–
Age	58 (R 31–78; IQR 47–73)	66 (R 41–79; IQR 57.8–72)	0.117
Sex (*M*)	9 (36.0%)	27 (61.4%)	0.076
PD duration (years)	–	3 (R 1–9; IQR 1–5.25)	–
Hoehn and Yahr score	–	2 (R 1–4; IQR 1.5–2.5)	–
MMSE score at baseline	29 (R 27–30; IQR 28–30)	27 (R 20–30; IQR 25.8–30)	0.009
MMSE score at follow-up	28 (R 26–30; IQR 27–30)	26.5 (R 17–30; IQR 23.8–29)	0.004
MoCA score at baseline	28 (R 27–30; IQR 25.5–28.5)	25.5 (R 17–30; IQR 22.8–28)	0.025
MoCA score at follow-up	26 (R 20–29; IQR 24–27)	22 (R 10–28; IQR 18.75–25)	<0.001
Follow-up duration (years)	4 (R 2–7; IQR 3–5)	2 (R 1–7; IQR 2–6)	0.197

*P-values, count, and percentages for sex and medians, ranges (R), interquartile ranges (IQR) for the other variables*.

### CSF biomarkers in diagnostic groups

**Figure 1:**
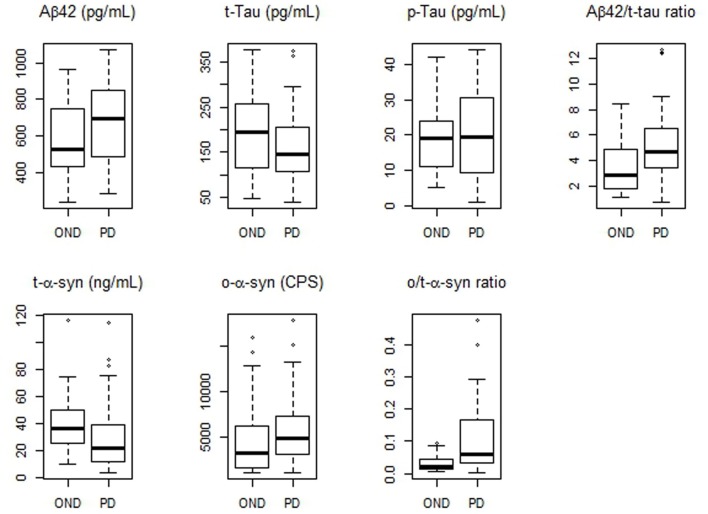
**Boxplots of CSF biomarkers values observed in PD and OND cohorts**. The horizontal bold lines indicate the medians; the lower part of boxes indicates the first quartile and the upper part the third quartile; the dashed vertical lines indicate the range of values.

Values of CSF biomarkers showed substantial overlap between the two groups (Table 2 and Figure 1). Although the differences did not reach the statistical significance, in PD group median Aβ_42_ levels were higher as opposite to lower median t-tau levels. As a consequence, Aβ_42_/t-tau ratio was significantly increased in the PD group with respect to OND subjects (*p* < 0.01, Table 2). Analogously to previous observation (Balducci et al., 2007), a significant decrease of t-α-syn (*p* = 0.015) and an increase of o-α-syn levels (*p* = 0.041) were found in PD group (Table 2). Interestingly the o/t-α-syn ratio greatly improved the discrimination between PD and OND groups (*p* < 0.001, Table 2). ROC analysis showed a sensitivity of 0.82 and a specificity of 0.56 for Aβ_42_/t-tau ratio. T-α-syn had a sensitivity of 0.59 and a specificity of 0.80. O-α-syn disclosed a sensitivity of 0.89 and a specificity of 0.48. O/t-α-syn ratio reached the best diagnostic performance having a sensitivity of 0.82 and a specificity of 0.64.

**Table 2:** **CSF biomarkers in PD and OND**.

	**OND**	**PD**	***p*-value**	**AUC**	**Sens**	**Spec**	**cut-off**
Aβ_42_	530 (431–752)	693 (493–852)	0.057	0.64 (0.51–0.78)	0.59	0.72	636.00
t-tau	194 (117–257)	146 (109–204)	0.085	0.63 (0.48–0.77)	0.64	0.68	159.00
p-tau	19 (11–24)	19.5 (9.75–30.25)	0.793	0.52 (0.38–0.66)	0.36	0.80	25.50
Aβ_42_/t-tau ratio	2.85 (1.88–4.88)	4.70 (3.47–6.38)	0.004	0.71 (0.59–0.84)	0.82	0.56	3.15
t-α-syn	36.5 (25.8–49.6)	22.15 (11.86–38.64)	0.015	0.68 (0.55–0.81)	0.59	0.80	24.45
o-α-syn	3139 (1500–6140)	4838 (3049–8141)	0.041	0.72 (0.59–0.84)	0.89	0.48	2565.50
o/t-α-syn ratio	0.021 (0.014–0.043)	0.061 (0.034–0.175)	<0.001	0.78 (0.67–0.89)	0.82	0.64	0.03

*Median values with interquartile ranges (IQR); ROC analysis summary with AUC (95% CI), sensitivity and specificity*.

In Table 3 the correlation analysis for all the CSF biomarkers considered is reported. Interestingly, in the PD group, an inverse association between t-α-syn and t-tau was found. Such a negative correlation was also observed in the OND group, where it did not reach the statistical significance. As expected, in the OND group a significant positive association between t-α-syn and Aβ_42_/tau ratio was observed.

**Table 3:** **Spearman's rank correlation matrix for CSF biomarkers in PD and OND groups**.

		**Aβ_**42**_**	**t-tau**	**p-tau**	**Aβ_**42**_/tau ratio**	**t-asyn**	**o-asyn**	**o/t-asyn ratio**
PD	Aβ_42_	1.00						
	t-tau	0.07	1.00					
	p-tau	0.25	0.71^***^	1.00				
	Aβ_42_/t-tau ratio	–	–	−0.44^**^	1.00			
	t-α-syn	−0.25	−0.33^*^	−0.25	0.13	1.00		
	o-α-syn	−0.12	−0.15	−0.14	0.07	0.06	1.00	
	o/t-α-syn ratio	0.15	0.11	−0.01	−0.02	–	–	1.00
OND	Aβ_42_	1.00						
	t-tau	0.28	1.00					
	p-tau	0.11	0.56^**^	1.00				
	Aβ_42_/t-tau ratio	–	–	−0.41^*^	1.00			
	t-α-syn	0.24	−0.25	−0.24	0.43^*^	1.00		
	o-α-syn	0.01	−0.14	−0.08	0.04	0.21	1.00	
	o/t-α-syn ratio	−0.34	−0.03	−0.04	−0.32	–	–	1.00

* p < 0.05,

** p < 0.01,

*** *p < 0.001*.

Table 4 reports the correlation analysis between CSF biomarkers and clinical parameters in OND and PD groups. In PD t-tau was positively correlated with the H&Y stage, as opposite to the Aβ_42_/t-tau ratio, which was inversely related to H&Y. Cognitive changes along time were measured as points lost in MMSE and MoCA scores between baseline and follow-up visits. In PD Aβ_42_ was negatively correlated with decline in MMSE and MoCA scores. Aβ_42_/t-tau ratio was negatively correlated with decrease in MMSE score. In OND group no significant correlation was found between CSF parameters and decrease in MMSE and MoCA scores.

**Table 4:** **Spearman's rank correlations between CSF biomarkers, age, disease duration, and clinical scores in PD group**.

		**Age**	**Disease duration**	**Hoehn and Yahr scale**	**MMSE score decrease**	**MoCA score decrease**
PD	Aβ_42_	−0.28	−0.13	−0.19	−0.52^***^	−0.45^**^
	t-tau	0.31^*^	0.04	0.39^**^	0.18	0.08
	p-tau	0.24	0.10	0.09	−0.10	−0.16
	Aβ_42_/t-tau ratio	−0.42^**^	−0.11	−0.50^***^	−0.37^*^	−0.26
	t-α-syn	0.00	0.30	0.10	0.06	−0.03
	o-α-syn	−0.26	−0.17	−0.14	−0.07	−0.07
	o/t-α-syn ratio	−0.18	−0.27	−0.12	−0.02	0.05
OND	Aβ_42_	−0.37			−0.01	−0.38
	t-tau	0.18			0.17	0.09
	p-tau	0.18			−0.26	0.26
	Aβ_42_/t-tau ratio	−0.43^*^			−0.12	−0.24
	t-α-syn	−0.41^*^			−0.37	−0.07
	o-α-syn	−0.02			−0.20	−0.41
	o/t-α-syn ratio	0.33			0.02	−0.24

* p < 0.05,

** p < 0.01,

*** *p < 0.001*.

### Multiple biomarkers evaluation in diagnostic groups

In order to assess the diagnostic performance of multiple biomarkers combination a logistic regression approach was used. Table 5 shows a summary of the best model according to several measures of test effectiveness, including sensitivity and specificity, positive and negative predictive values, positive/negative likelihood ratio, AUC and diagnostic odds ratio (DOR). The model included Aβ_42_/t-tau and o/t-α-syn ratios, which together reached a specificity of 84% and a sensitivity of 70%.

**Table 5:** **Logistic regression analysis of multiple CSF biomarkers between PD and OND**.

	**Estimate**	***SE***	***p*-value**	**Accuracy measures**	
Intercept	−2.540	0.882	–	Sens = 0.70	LR+ = 4.40
Aβ_42_/t-tau ratio	0.405	0.162	0.012	Spec = 0.84	LR− = 0.35
o/t-αsyn ratio	28.514	11.124	0.010	PPV = 0.89	DOR = 12.52
				NPV = 0.62	AUC = 0.82 (95%CI = 0.73–0.92)

**Figure 2:**
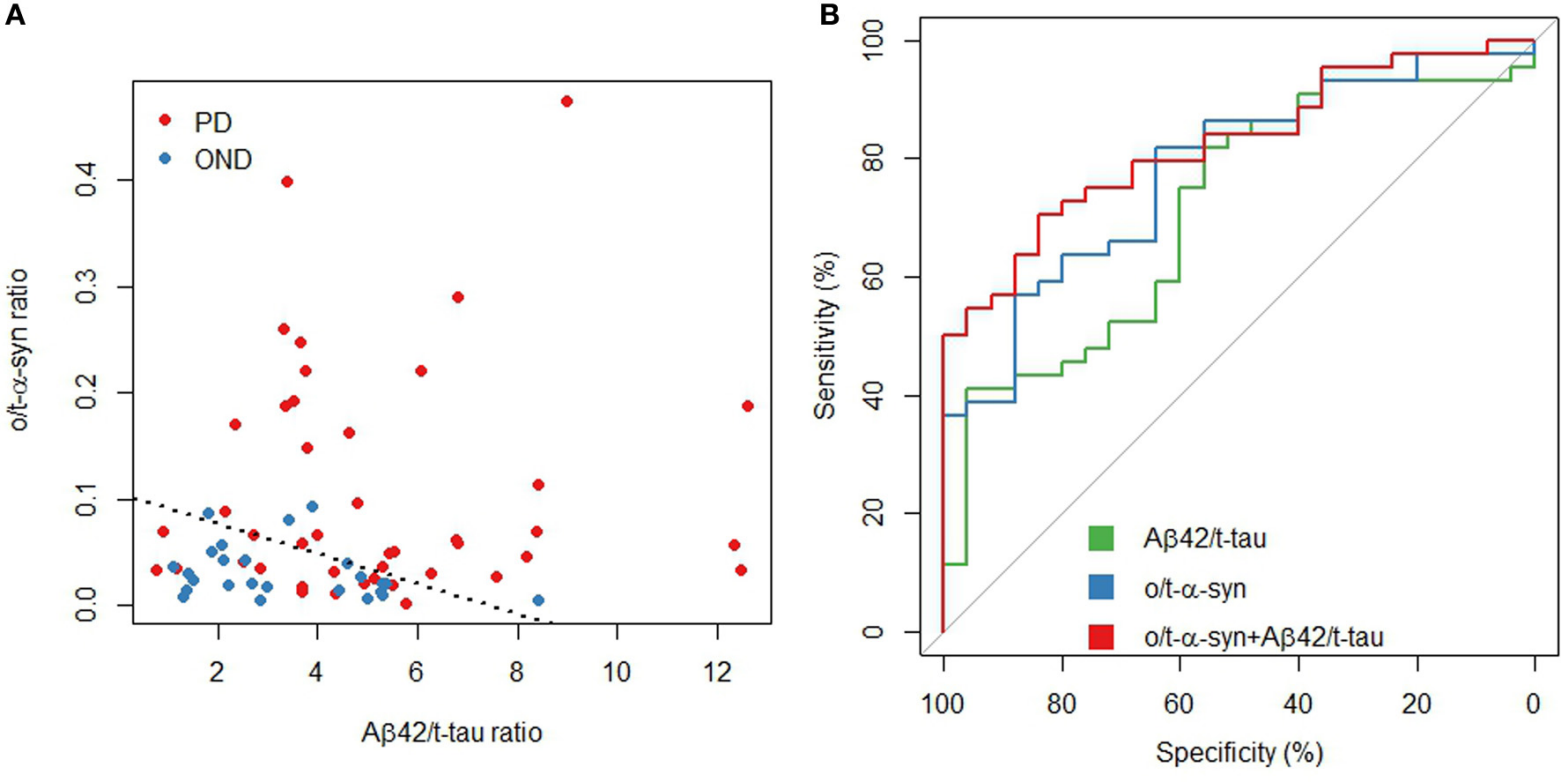
**Aβ_42_/t-tau and o/t-α-syn ratios in PD and OND. (A)** Scatterplot: the dashed line represents a partition of the o/t-α-syn and Aβ_42_/t-tau space such that below the line the model predicts OND, above the line the model predicts PD **(B)** ROC curves of Aβ_42_/t-tau and o/t-α syn ratios and the fitted values of the multivariable logistic regression model.

Figure 2 shows how the model separates PD patients from OND, allowing for a good discrimination of PD and how the model predictions reach a superior diagnostic performance with respect to the o/t-α-syn or Aβ_42_/t-tau ratios, separately.

### CSF biomarkers for predicting cognitive decline in PD

As reported in the previous section, Aβ_42_ and the Aβ_42_/t-tau ratio were the only parameters showing a correlation with MMSE and MoCA scores. To investigate the relationship between the decrease of these two neuropsychological measurements with Aβ_42_ and Aβ_42_/t-tau ratio, a multivariate linear regression model was applied, adjusting for the baseline values and follow-up duration (Table 6). MMSE score decrease confirmed to be significantly associated with low CSF Aβ_42_ levels at baseline; the same trend was also observed for MoCA scores; Aβ_42_/t-tau ratio was not significantly associated with cognitive decline.

**Table 6:** **Linear regression analyses of cognitive decline in PD cohort**.

		**Estimate**	***SE***	***p*-value**
MMSE score decrease	Intercept	3.60	–	–
	1/Aβ_42_	1139.92	329.08	0.012
	MMSE score at baseline	−0.17	0.08	0.038
	Follow-up duration (years)	0.18	0.08	0.029
MoCA score decrease	Intercept	2.75	–	–
	1/Aβ_42_	1395.25	482.93	0.007
	MoCA score at baseline	−0.08	0.09	0.387
	Follow-up duration (years)	0.22	0.11	0.048

## Discussion

As in other neurodegenerative disorders, PD is characterized by a large time gap between the beginning of neurodegenerative processes and the onset of clinical neurological manifestations. The disease's natural history includes a first asymptomatic stage, followed by a long pre-motor phase; finally, when the classical motor symptoms appear, the majority of nigral dopaminergic neurons are already affected by degeneration. The classical diagnostic criteria for PD mostly rely on motor symptoms, making the formulation of an early diagnosis very challenging. Another challenge for the research focused on this disorder is the understanding of the mechanisms underlying the development of dementia taking place in a subgroup of parkinsonian patients. It would be very important to have the possibility to individuate those patients at risk to develop this devastating complication to initiate possible protective pharmacological and non-pharmacological interventions. Thus, the availability of objective measures such as reliable “biomarkers,” indicators of biological/pathogenetic processes, will be of great importance both for diagnostic accuracy and prognostic evaluation.

In this context, CSF analysis might be of great importance since CSF dynamically reflects the pathophysiological processes taking place in the brain. At present, CSF biomarkers are a routine analysis for early diagnosis of AD. Accordingly, increasing interest is focused on CSF biomarkers in PD, with major expectations on α-syn species and other misfolding proteins, namely β-amyloid and tau. With respect to diagnostic performance, data available so far indicate that there is not a unique ideal CSF biomarker, rather the combination of molecules related to different pathophysiological pathways involved in PD may represents a good strategy for obtaining a more accurate diagnosis (Parnetti et al., 2014). Concerning the prediction of cognitive decline in PD, the most consistent role as predictive factor is played by low CSF Aβ_42_ levels (Parnetti et al., 2008; Alves et al., 2010; Siderowf et al., 2010; Leverenz et al., 2011) although also tau species have been postulated to represent prognostic factors (Zhang et al., 2013). Interestingly, a recent investigation (Kang et al., 2013) carried out in drug-naïve patients with early PD, showed slightly lower CSF levels of both t-tau and t-α-syn in PD compared to healthy controls. This finding offered the Authors the opportunity to speculate that the interaction between tau proteins and α-syn may limit the release of tau proteins into CSF.

In this investigation we assessed both the diagnostic accuracy and the performance in predicting cognitive decline of the combination of CSF AD biomarkers (Aβ_42_, t-tau, p-tau, and Aβ_42_/t-tau ratio) and α-syn species (t-α-syn, o-α-syn, and o/t-α-syn ratio) in a cohort of PD patients and neurological controls followed up for 2–6 years (median follow-up duration: 3 years).

With respect to the diagnostic performance of the biomarkers considered, none of them demonstrated acceptable values in terms of sensitivity and specificity when taken separately. Aβ_42_/t-tau and o/t-α-syn ratios showed good sensitivity (0.82) but low specificity (0.56 and 0.64, respectively). While the usefulness of o/t-α-syn ratio in discriminating PD and controls has already been reported in recent investigations (Tokuda et al., 2010; Park et al., 2011; Sierks et al., 2011; Parnetti et al., 2014), the Aβ_42_/t-tau ratio deserves some comments. Interestingly, in the PD group, we found slightly higher values of Aβ_42_ together with lower values of t-tau as compared to OND group. As a consequence, the mean value of Aβ_42_/t-tau ratio was significantly higher in PD patients with respect to the OND group. This may be due to the fact that our control group was not including healthy subjects, being composed by patients with other neurological diseases. Interestingly, reduced CSF Aβ_42_ levels at baseline represented a predictive factor for cognitive decline only in the PD group. In fact, only in PD patients lower CSF Aβ_42_ levels were correlated to a more marked decrease in MMSE and MoCA scores at follow-up. The finding of reduced CSF Aβ_42_ levels in PD patients is quite controversial, being reported in some (Sjögren et al., 2002; Zhang et al., 2008; Alves et al., 2010) but not in other papers (Pøikrylová Vranová, 2010; Siderowf et al., 2010; Leverenz et al., 2011).

A clear positive association was also observed between t-tau and t-α-syn in PD group. These findings are consistent with the observation of Kang and coworkers, describing the occurrence of lower CSF levels of t-tau and t-α-syn in PD patients. A reasonable explanation may be the mutual interaction of the two molecules, leading to a reduced release of tau in CSF. The discriminative power between PD and OND significantly improved when considering both o/t-α-syn ratio and Aβ_42_/t-tau ratio, as shown by the logistic regression analysis, and further illustrated in Figure 2. This confirms that the combination of several biomarkers is more helpful than single biomarkers for adding diagnostic accuracy of PD.

About the predictive value of CSF biomarkers for cognitive decline in PD, our study confirmed the specific role of low CSF levels of Aβ_42_ in this pathological condition; no other biomarker was significantly associated to this outcome measure. For assessing cognitive function along time, we used both MMSE and MoCA (Gill et al., 2008). Both neuropsychological instruments showed to be related to CSF Aβ_42_ levels, i.e., lower the CSF Aβ_42_ levels, greater the decrease in MMSE and MoCA scores. The same holds true for Aβ_42_/t-tau ratio with respect to MMSE. Multivariate analysis (adjusting for follow-up time and baseline measurements) confirmed that low CSF Aβ_42_ levels are independent predictor of cognitive decline in PD, either measured by MMSE or MOCA.

In conclusion, this study further contributes to the evidence of the usefulness of CSF biomarkers for PD diagnosis and prognosis. Major points are the need to combine several CSF biomarkers for improving the diagnostic accuracy, and the confirmed role of low CSF Aβ_42_ levels as independent predictor of cognitive decline in PD. Longitudinal studies measuring biomarkers and clinical parameters over several years represent a major contribution in this field. Analogously to the longitudinal AD Neuroimaging Initiative (ADNI, http://adni-info.org/) in AD, the Parkinson's Progression Markers Initiative (PPMI, http://ppmi-ifo.org/) will give important knowledge in the field of PD, thanks to the measurement of several CSF, blood, and imaging biomarkers in early *de novo* PD followed up for several years.

### Conflict of interest statement

The authors declare that the research was conducted in the absence of any commercial or financial relationships that could be construed as a potential conflict of interest.
